# Form and Function: Learning Anatomy Using Ultrasound

**DOI:** 10.1007/s40670-023-01806-y

**Published:** 2023-07-03

**Authors:** Janine C. Correia, Ilse Meyer, Lakshini McNamee

**Affiliations:** 1 Division of Clinical Anatomy, Faculty of Medicine and Health Sciences, Stellenbosch University, P.O. Box 241, Cape Town, 8000 South Africa; 2 Centre for Health Professions Education (CHPE), Faculty of Medicine and Health Sciences, Stellenbosch University, Cape Town, South Africa; 3 Education Development Unit, Faculty of Health Sciences, University of Cape Town, Cape Town, South Africa

**Keywords:** Ultrasound, Anatomy education, Instructional design, clinical relevance, Integration, Spatial learning

## Abstract

Ultrasound (US) is increasingly used across medical specialities as a diagnostic tool and medical faculties are therefore further incorporating imaging into their programmes. Using US within undergraduate instruction has several benefits. US, as a learning instrument, may strengthen existing anatomical knowledge and improve visual understanding of anatomy. The cost-effectiveness, as well as portability of the US, makes it a valuable means to add to traditional anatomy teaching modalities. Furthermore, students have an opportunity to develop skills in interpreting US images and this may add a different element to the learning of anatomy. This study aimed to explore undergraduate clinical anatomy students’ perceptions of the use of US as an add-on to cadaveric dissection. Students were invited to participate in virtual focus group discussions. Three virtual focus group discussions were conducted, and 11 participants volunteered to take part. Thematic analysis of the data generated six themes. These are described as *the study of living anatomy*, *learning cross-sectional anatomy*, *enhanced relevance of anatomy learning*, *increased interest in anatomy*, *instructional design*, and *the affective and technical experience of using US*. The results suggest that it is feasible and advantageous to implement US sessions as an add-on to the teaching of anatomy during practical dissection sessions of clinical anatomy students. The use of innovative technologies such as US enhances the interest of students and allows them to develop dexterity and competencies in their learning process.

**Supplementary Information:**

The online version contains supplementary material available at 10.1007/s40670-023-01806-y.

## Introduction

Anatomy is an important module in the curricula of many health professions and has been a cornerstone in medical education throughout history [1–4]. Furthermore, anatomy is a unique, visual, high content-based, and practical course subject, which involves investigating and understanding three-dimensional anatomical structures. There has been a shift from passive, instructive, and extremely exhaustive anatomy courses towards clinically relevant anatomy courses [1]. Consequently, it is especially important to find innovative ways to teach such a highly content-based subject while integrating clinical anatomy.

Anatomy is a challenging subject to teach and learn. Firstly, anatomy includes numerous new concepts, making it a perplexing subject [5]. Secondly, current trends in health professions education include decreased teaching time for anatomy [2, 4, 6]. Thirdly, it is challenging to find and evaluate methods to instruct a diverse group of students more efficiently, especially with limited resources. Lastly, modern health professions education has moved its focus from learning fundamental medical sciences in compartments, towards integrated clinically applied anatomy [7].

Clinical anatomy undergraduates spend a substantial number of hours in dissection halls and practical classes studying anatomy using prosections or plastic and plastinated anatomical models, or by dissection during the undergraduate period [8]. However, purely covering the content in anatomy objectives with instructive lectures and practical dissection sessions may not be as effective for a long-term understanding of anatomy [5, 9]. Teaching and learning anatomy may benefit from various innovative teaching modalities, such as those offered by technological advances and the digital revolution. Utilizing anatomical models, enhancing clinical relevance, body painting, and advanced imaging technologies have been described [1]. Strategies that encourage interest in anatomy should be advocated and interventions that assist students to develop a better understanding of living and clinical anatomy should be pursued.

Imaging technologies can be used to observe normal and anatomical pathologies and variations [4]. Radiographs allow students to observe skeletal anatomy, while US images permit students to visualize soft tissues and organs in real-time, which can be used to complement cadaveric dissection [4]. Alternatively, computerized tomography scans and magnetic resonance imaging assist particularly in the study of sectional anatomy, by transforming three-dimensional (3D) organs and structures into a two-dimensional (2D) layout [4, 9].

It is clear from these studies that anatomy learning remains a substantial part of the foundational sciences that must be successfully mastered. US has the potential to offer a connection between students’ comprehension of anatomy and their subsequent evaluation of actual patient anatomy in clinical practice. According to Maher and Hale [10], US of the musculoskeletal system used during the teaching and learning of anatomy “brings anatomy to life” and highlights the clinical significance of why it is relevant to the students.

This study reported on the explored undergraduate clinical anatomy students’ perceptions of using US as an instructional strategy. Thematic analysis and interpretive findings contributed to a better understanding of how undergraduate students experience US for the learning of anatomy.

## Materials and Methods

### Context

A qualitative case study design within the interpretive/constructivist paradigm was followed to explore students’ perceptions of the use of US in teaching and learning anatomy. The study occurred at the Division of Clinical Anatomy, Faculty of Medicine and Health Sciences, Stellenbosch University, and included all undergraduate, third-year clinical anatomy students (25 students) enrolled for a BSc Human Life Sciences degree. The sampling approach for the evaluation of the use of US in teaching and learning anatomy was a convenient one. Eleven (11) participants volunteered and signed the written consent form.

### Ultrasound Session Instructional Design

**Fig. 1:**
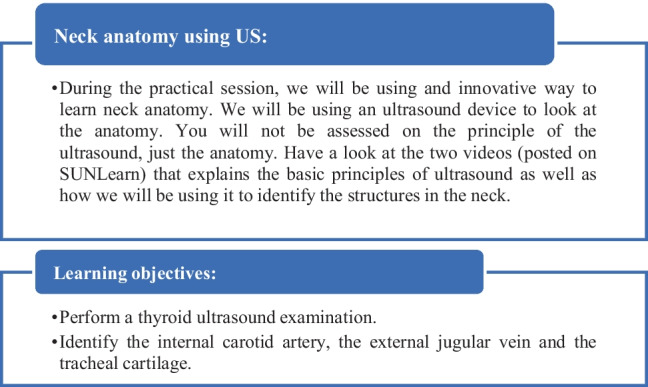
Learning objectives of the US session

US sessions were incorporated during routine dissection/practical sessions of the neck region. Students in the clinical anatomy undergraduate programme mostly use cadaveric dissections and the use of prosections to aid them in their anatomy learning. The anatomy of the neck and thyroid was used as most anatomical structures can be visualized easily and students can perform the ultrasound on themselves [3]. The hands-on session objectives (Fig. 1) were developed in advance and aligned with the outcomes of the anatomy dissection sessions. The learner-centred, instructional design model for the US session followed the PLHET model (Table 1) of preparation, linking, hooking, engagement, and transfer as described by Jurjus et al. [11].

**Table 1:** Instructional design based on the PLHET model

**Session component**	**Purpose**	**Ultrasound session**
**Preparation**	Provide learners with background information and set expectations	Virtual lectures, videos, and outcomes were posted on SUNLearn before the session
**Linkage**	Stimulate students’ learning: link what is to be learned to what learners already know and/or have experienced	Reference to the information from the anatomy lecture session
**Hook**	Stimulate students by showing the significance of the material to their work	Clinical scenarios (thyroid biopsy and pathologies) were discussed during the session
**Engagement**	Have students apply the material, integrate it with their prior knowledge/skills, and generate new knowledge/skills	The demonstration was done by the instructor using the ultrasound device (Versana Active)
**Transfer**	Strengthen retaining of recent learning by way of having students apply it to a new situation	Students used the ultrasound device themselves and identified the thyroid gland, common carotid artery, and the external jugular vein

A pre-scanned colleague in the Division of Clinical Anatomy volunteered and gave consent to be scanned [12]. The students used the US device to scan themselves as well as the pre-scanned colleague. Two labelled diagrams, a transverse, and a longitudinal/sagittal section of the neck were placed at the US station. The labelled diagrams included an illustration of the placement of the probe and the subsequently labelled transverse and sagittal images produced by probe placement.

### Data Collection and Analysis

Students were invited to participate in virtual focus group discussions on Microsoft Teams^™^. Each virtual focus group discussion lasted approximately 60 min. Three focus group sessions were conducted and consisted of 3–5 students. Ten questions were posed to each group, which guided the interviews (SI 1). Data recording of the focus groups was done through voice recording on Microsoft Teams. Notes were made immediately after the virtual focus group sessions by the researcher. The thematic analysis of the data obtained from the focus groups was conducted by using the six steps of Creswell: (1) organizing and preparing the data, (2) reading through all the data, (3) coding the data, (4) describing and identifying themes, (5) presenting the findings, and (6) interpreting the data [13].

## Results

The thematic analysis of the transcribed data identified six main themes and sub-themes. All participants were labelled according to focus group, and gender (for example, P1MFG1 would be participant 1, male, focus group 1). Table 2 shows the six main themes and sub-themes generated from the participants’ responses. Responses are included in italics in text boxes as direct quotations to reflect participants’ answers.

**Table 2:** Main themes and sub-themes

**Themes**	**Subthemes**
1. **The study of living anatomy using the US**	• Comparison between US and cadaveric dissection • Functional/moving anatomy • Surface anatomy
2. **US and learning cross-sectional anatomy**	• Dimensional learning • Textbook comparison
3. **Enhanced relevance of anatomy learning**	• Applied anatomy • Clinical relevance
4. **Increased interest in anatomy after the US sessions**	• Motivation to learn • Memory/retention
5. **Instructional design of the US session**	• Labelled US diagrams • Pre-ultrasound material • Instructional format and group size
6. **The technical and affective experience of using the US device**	• Introduction to the US equipment and sonogram interpretation • Excitement to use the US device • Hands-on experience using a US device

### The Study of Living Anatomy Using US

US enabled visualisation of the action of anatomical structures and the dynamic state of human anatomy in relation to surface landmarks; elements that might be difficult to grasp in the traditional setting of the dissection hall with cadaveric specimens. This theme describes students’ perceptions of how US as an imaging instrument had enabled them to experience living anatomy. The sub-themes that were relevant *compared US to cadaveric dissection*, *described functional/moving anatomy*, and *learning of surface anatomy.*

#### Comparison Between US and Cadaveric Dissection

Participants compared using US to the dissection of a cadaver. Students felt that US was less laborious and less tedious. They described how the structures could be observed in real-time, in a non-invasive manner, and without dissecting anything. However, participants felt that they would prefer a combination of US and cadaveric dissection, rather than using a single modality. The cadaveric dissections added meaning to and enhanced their understanding of the images seen in the US.

*Especially, it was very interesting to know that although you're not actually cutting the body, you can still like get a transverse section and a longitudinal section. P4FG1M*

*Or you always think you must cut a specimen, so you'll need a donor body to do it. But now with an ultrasound you can view it in real-time. P4FG1M*

*I think if you have a dissection before the ultrasound and then look at the ultrasound structures ... P7FG2F*

#### Functional/Moving Anatomy

Functional anatomy refers to the study of anatomy concerning function, and several participants reported that it was beneficial to view this in a living person. The dynamic element that US added to their learning experience, that cadaveric dissection cannot achieve, motivated learning by bringing anatomy to life.

*Living anatomy is so interesting because it's dynamic and you can see exactly what's going on [using the US] and how things are moving and working. P11FG3F*

#### Surface Anatomy

Surface anatomy is the study of the exterior features of the body, specifically concerning its interior parts. Participants commented on how using US added to their understanding of the visual surface anatomy and linked it to the deeper anatomy.

*You learn to look for landmarks and you can link the surface anatomy of the neck to the internal structures as well. P7FG2F*

### US and Learning Cross-Sectional Anatomy

Cross-sections are two-dimensional views of gross anatomical structures in the transverse planes. Thus, a solid understanding of the spatial relationship of anatomical structures is essential in anatomy learning. Two sub-themes were identified in this theme namely, *dimensional learning* and *textbook comparison.*

#### Dimensional Learning

Undergraduates frequently experience difficulties in their spatial understanding of 3D anatomy from 2D images, for example, images found in anatomy textbooks and on the internet. Participants felt that the US helped them see the various layers of skin, tissue, and muscle, which helped them visualize a different dimension compared to cadaveric specimens where the layers were already removed. US presented a reliable approach to understanding the spatial relations between anatomical structures.

*Improving our understanding of it [cross-sections] and seeing it from a different perspective. P6FG2F*

*I think when we do cross-sectional anatomy with the cadaveric specimens; we often only look at one cross-section in isolation. We never look at the layers. P8FG2F*

*I think that the cross-sectional view helps a lot with getting the relations between the midline and what lies laterally. P3FG1M*

#### Textbook Comparison

Textbook diagrams were viewed as difficult to interpret as pictures represent a single and static view of complex anatomical structures; consequently, how the structures positionally relate to each other can be difficult to visualize from these diagrams. The participants perceived the US image to be more realistic and static depictions from textbooks were most likely to make more sense to students having viewed the same cross-sectional image from the US. Most respondents found the US device a valuable aid in improving their understanding of three-dimensional anatomy.

*I think that it helped me visualize the structures in relation to one another, especially in the cross-section. I think it's quite difficult to see how things lie in relation when you're just looking at a picture. P10FG3F*

*Think a lot of textbooks tend to make structures look similar when they illustrate how things look. Whereas in the ultrasound, I was surprised to see that they do look very different. P5FG1M*

### Enhanced Relevance of Anatomy Learning

The US session connected the importance of anatomy to the clinical field. Furthermore, the participants also applied the new knowledge to different modules, research projects, and postgraduate studies. This theme includes all the responses relating to ultrasound being potentially helpful for integrating anatomy with clinical practice or postgraduate studies, or applied to other aspects of anatomy. The two subthemes in this theme were *applied anatomy* and *clinical relevance*.

#### Applied Anatomy

Participants had considered introducing US in their research projects or had reflected on how the enhanced clinical relevance they experienced with the use of US may also be applied in their postgraduate studies.

*I was also thinking about my anatomy [research] proposal and trying to find things [applications]… using the ultrasound…P8FG2F*

*Also, to connect your undergraduate [anatomy] to your postgraduate [anatomy] to see it clinically and to apply your knowledge in a sense. P6FG2F*

#### Clinical Relevance

During the US session, a clinical scenario, thyroid biopsy, was discussed by the facilitator to stimulate learning by showing the relevance of the US anatomy to the clinical setting. Although the participants are non-medical students, they perceived the value of the US session in bridging the gap between the basic sciences and the clinical significance of learning anatomy. A participant also suggested that a clinician might be brought in to make the session more meaningful.

*Ultrasound is that perfect link between the two; connecting the textbook notes to what you actually see in the clinical field. And I think definitely this is something that all medical professionals (whether you study Anatomy or medicine or anything like that) will benefit from using to connect the textbook to the actual clinical practice. P7FG2F *

*I think bringing in a clinician would increase the interest that students feel… P3FG1M*

### Increased Interest in Anatomy After the US Sessions

Students found the US session influenced their affect even if their test scores might not be increased; they were more interested and motivated to learn anatomy. Two sub-themes were found in the theme of increased interest in anatomy; *motivation to learn* and *memory retention*.

#### Motivation to Learn

US activities promoted students’ motivation to learn anatomy; the novelty of the teaching strategy promoted their interest to engage with resources and they felt that they would remember the content better.

*It was very new but with that excitement and like that newness also comes a very large interest aspect. So, I feel like I was more interested in learning about that area and going back to my textbook. P4FG1M*

#### Memory Retention

One of the challenges encountered by many anatomy lecturers is how to assist students to learn and then recall a large volume of facts. Consequently, some participants commented that it was easier to learn anatomy following the US session and they felt that they remembered the anatomical structures better. The US sessions assisted with spatial cognizance, leading to improved student engagement, understanding, and retention of anatomical concepts.

*It was easier to study afterwards now that I had seen it and it was easier to go back to the textbook and study it. Then I found that my memory retention was greater because I saw it like that. P6FG2F*

*So, adding different things to your study methods, so the cadaver with images with ultrasound, gives you all the different views of something and for me, it will help me remember things in the long run. P10FG3F*

### Instructional Design of the US Session

Before the US session, the introduction to basic ultrasound physics and knobology was shared with the students. In addition, at the US station, labelled sonograms of the neck region were placed to help the students with the identification of the anatomical structures. A demonstration was given in small groups and later students were encouraged to use the US device or technology themselves. Connecting the US session to the relevant lecture material and motivating students to be active participants in their learning made the session more purposeful for them. The categories in this theme include *pre-ultrasound material*, *labelled US diagrams*, and *instructional format*.

#### Pre-Ultrasound Material

Pre-ultrasound material was made available before the session. The participants had mixed opinions of the material. Some participants felt that the videos were informative and other participants felt that the explanation the researcher gave before commencing the US session was more helpful than the online videos.

*It was very useful. The video that was actually speaking about how to do the ultrasound was very useful and very informative. And the other video where it implemented the physics side of things; was nice to have that kind of background. P11FG3F*

*The explanation we got at the actual ultrasound session was very helpful in understanding the actual protocol behind performing the ultrasound. P8FG2F*

#### Labelled US Diagrams

Two labelled diagrams were placed at the US station depicting a longitudinal and transverse image of the neck as well as probe placement to obtain specific US images. Participants viewed the labelled US diagrams as effective in aiding them to identify the anatomical structures. Thus, the use of cross-sectional images, together with line drawings, could support the sonogram orientation of anatomical structures during US sessions.

*I think that because there were reference pictures on the wall, it made it a lot easier because it was labelled on the picture, and we were seeing the image in front of us while we were doing the ultrasound. P10FG3F*

#### Instructional Format and Group Size

The class consisted of 25 students, and students were encouraged to form groups of six to observe the demonstration of the device, as well as use it themselves. Participants commented negatively regarding the group size at the US station; they suggested overcoming this by projecting US images onto a large screen. Participants, however, valued the opportunity to experience using the novel technique to study anatomy and appealed for more practical sessions with the US.

*When everyone was looking at the ultrasound and standing around the table, then it's difficult to see the pictures [US image] sometimes, so just smaller groups. P2FG1F*

*I think it would be nice to see things [anatomy systems] through ultrasound like when we are doing musculoskeletal as well, just to see where the muscles lie in relation to one another. P11FG3F*

### The Technical and Affective Experience of Using the US

The participants were introduced to the US equipment for the first time during the practical session and were excited to use the US device to learn anatomy and to use the equipment themselves, even though they were uncertain in the beginning. The subthemes include the *introduction to US equipment*, *sonogram interpretation*, *the excitement of using the device*, and* their hands-on experiences of using it.*

#### Introduction to the US Equipment and Sonogram Interpretation

Participants often describe difficulty in interpreting US images; they found it difficult to identify familiar anatomy structures when illustrated in grayscale, particularly when changing the orientation of the probes. Thus, participants mentioned that it took them a while to get used to interpreting the US image on the screen. Most participants struggled to identify the structures because of the grayscale of the sonogram but commented on the value of turning on the color mode on the device to assist them with identifying the structures.

*So initially, it was a bit confusing, but once I got the hang of exactly how we are looking at it and how the images are being made, then it became a lot easier to see what is going on. P5FG1M*

*It was very difficult in the beginning to orientate yourself because it is in black and white [grey scale] and it looks very different from what we used to [in the cadaver], but once we could find the landmark and orientate ourselves, I found it was a lot easier. P6FG2F*

#### Excitement to Use the US Device

The participants were enthusiastic to use the US and there was excited anticipation in the classroom before using it. The use of US was perceived as an innovative, stimulating, and engaging manner to stimulate learning of living and clinical anatomy.

*It was also quite exciting to use ultrasound. You know days before, we were excited. P6FG2F*

#### Hands-on Experience Using the US

The students were encouraged to use the device to scan themselves and each other. A few participants also commented on the hand–eye coordination of using the US and how the hands-on experience of matching their hands with the screen and incorporating that knowledge into learning anatomy.

*And we could do it ourselves, hands-on and look at different students’ neck regions and see maybe if there was variation. P10FG3F*

*On the one hand, you used your hand-eye coordination to try and orientate where you were on the neck, but while looking at the screen, it incorporates a lot more senses... P7FG2F*

## Discussion

### The Study of Living Anatomy Using the US

Living anatomy, defined as the anatomy of living humans, is gathering importance in contemporary anatomical science education [6]. Cadaveric dissection teaches the hand and eye, but then again, it does not show in what way human anatomy functions. To think of anatomical structures in terms of function, students must relate the cadaveric structures with the information they might find from the system and the function of these structures in the living body. Therefore, the dynamic nature of US allows the demonstration of movement and integration of structures within a living body, with visualization of the functional.

Traditionally, cadaveric dissection is the preferred pedagogical approach for teaching and learning anatomy [1, 6, 16]. Although dissection is perceived as the gold standard to teach and learn anatomy, research has also suggested that cadaveric dissection is not a standardized learning event [15, 16]. Dissection can be perceived by a small group of students as a poor learning strategy since students become emotionally afflicted and cannot engage in the activity [17].

Similarly, in this study, students appreciated the multimodal teaching approach by incorporating different elements in their learning, such as complementing the dissection session, traditional atlas, and didactic lectures with the use of US. Bullen et al. found that the multimodal learning approach, the add-on of the US sessions to the anatomy module, which included didactic lecture and practical sessions, supports groups of students with different preferences [18]. Moxham and Moxham described that using cadaveric dissection and medical imaging together advances students’ capability to recognize anatomical structures and offers long-term memory retention [19].

This study further confirms that studying living anatomy using US enhances understanding of how anatomical structures in living humans’ function and move in relation to one another in a way that cadaveric dissection lacks. The participants’ feedback that the US added value to better learning of living anatomy is supported by evidence in the literature [8, 12, 20, 21]. As a non-invasive and non-destructive way to see inside the living body, US is a perfect add-on to teach living anatomy [22]. However, many anatomical structures with intricate courses and relations are challenging to observe with US; dissection, although perceived as time-consuming, provides a haptic approach that cannot be achieved by ultrasound.

Surface anatomy and living anatomy are vital for “understanding the foundation of physical examination, and the interpretation of clinical findings” according to Azer [23]. Students found the US session helped their understanding of surface anatomy and this is echoed in similar studies in the literature [18, 24] and it provided a clearer view connecting surface anatomy with internal anatomy [11]. Although not much emphasis is placed on teaching surface living anatomy in textbooks according to Azer [23], US being included in anatomy practical sessions might change this in the future.

### US and Learning Cross-Sectional Anatomy

Gross anatomy serves to orientate students to the complex 3D nature and relationship of the structures within the human body. In this study, the participants perceived an improvement in 3D anatomical imagination. The ability to have a clear understanding of the spatial relationship of anatomical structures is an essential skill in anatomy learning [25]. According to Vorstenbosch et al., a good spatial ability is advantageous for learning anatomy and may be useful for students’ spatial comprehension [25].

Including innovative technologies as add-ons to dissection enables the observation of the system in vivo and advances students’ awareness of spatial relationships and the relationship between anatomical structures [26, 27]. Comparable with the literature, participants found that using the US had improved their understanding of spatial relationships of key anatomical structures [26, 28]. Furthermore, participants compared the cross-sectional US images with anatomical cadaver cross-sections and textbook depictions and found these had been valuable in adding to their comprehension.

Participants also perceived the cross-sectional view of the US to be more realistic than just looking at a static image. In the clinical setting, the goal is to generate an image that is as realistic as possible. Anatomy textbooks and atlases show a different reality compared with viewing anatomy structures in real-time in US [29]. Furthermore, US permits the visualization of anatomical structures and relationships—anatomical structures which are otherwise difficult to observe using static models or images [22].

### Enhanced Relevance of Anatomy Learning

One of the great concerns for medical and other health sciences students is connecting theoretical subjects with practical ones and their utilization in clinical practice. Many medical schools have experienced a need for transformation in instructional methods because of the high content volume, and because the basic sciences often seem disconnected from clinical practice [30]. However, from the feedback received, the US session connected the importance of anatomy to the clinical field [31, 32]. Stringer et al. found that using US as a method of teaching anatomy provides learners with an overview of the clinical value of US, and, by concentrating on anatomical outcomes rather than the acquisition of practical imaging skills, strengthens the learning of clinical anatomy [20].

The integration of clinical anatomy into traditional anatomy courses aids the understanding of anatomy and improves clinical thinking [33]. Additionally, the participants also applied their newly acquired knowledge of clinical anatomy and US training to several aspects of other modules and postgraduate studies. Thus, these interactive sessions could stimulate critical thinking skills in undergraduates [18] and play a valuable role in the integration of foundational sciences.

The current study focused on undergraduate clinical anatomy students’ perceptions. Many studies found in the literature focus on implementing US in the curriculum of UG medical students [12, 21, 30, 34, 35]. However, there is limited literature to support including US for basic science education students in non-medical degrees [18, 27, 36, 37]. Yet, two recent studies investigated the utility of US as an anatomical learning tool for Bachelor of Science students or non-medical graduate students [18, 27]. Encouraging non-medical students to become skilled in the pedagogical use of US technology will produce an innovative group of anatomy educators eager to implement US sessions to complement anatomy modules at universities [18]. Furthermore, US experience will make clinical anatomy graduates more competitive in the employment market and will be a valuable skill for individuals who intend to teach in a medical school [37].

### Increased Interest in Anatomy After the US Sessions

The results of this study gave insight into the question of whether teaching with innovative modalities, such as US, promotes and enhances interest in anatomy as a subject. The evidence from the present study, as in other studies [29, 31, 36], suggests that the participants had an increased interest and motivation to learn anatomy. According to Rodríguez-López et al., students who used imaging techniques to learn anatomy, developed a long-term positive perception of the subject [29]. Another study by Moscova et al. found that by incorporating imaging in practical sessions, student attendance increased for practical sessions; many students displayed interest in imaging technologies and were motivated to learn more [28].

This study suggests that hands-on teaching in US promoted meaningful learning and that students perceived that it had a positive impact on their memory retention of anatomical information; like a study conducted by Dreher et al. [31]. The real-world, hands-on nature of US assists students to acquire factual learning uniquely and this can motivate the development of episodic memory. Episodic memory, a category of long-term memory, is linked more closely to experiences and specific events, than to factual learning [38]. Learning strategies are vital to learning acquisition and memory retention, for example increasing time spent on activities and creating opportunities for constructive learning, including chances for collaborative learning [14].

### Instructional Design of the US Session

The learner-centered instructional design model for the US sessions followed the PLHET model of preparation, linking, hooking, engagement, and transfer [11]. This approach was based on similar sessions for undergraduate medical students. The US sessions were adjusted for clinical anatomy students; however, many aspects of the instructional design that were successful for medical students were also effective for clinical anatomy students. Bullen et al. used this design to include US to complement anatomy instruction in a non-medical course and participants found the sessions to be arranged efficiently, in a way that strengthened the course material [18].

The placement of supplemental online learning materials about the US before the session was aimed at improving the students’ preparation for the sessions [12]. However, some students noted that they did not study the online material and would have preferred the videos to have been played in the practical session immediately before the US session. To maximize the learning potential of the US session, the students needed to be prepared with the relevant information and a basic understanding of knobology relating to US.

Furthermore, participants viewed the labelled US diagrams at the US as effective in aiding them in identifying the anatomical structures. Similarly, in findings by Moscova et al., participants indicated more assistance was required in interpreting US images and participants suggested additional notes with labelled structures [28]. Participants initially found the sonograms difficult to interpret, as seen in theme 6, but found the labelled diagrams assisted them. Thus, the use of cross-sectional anatomy images and line diagrams together could aid in identifying structures during US sessions, similar to findings by Swamy and Searle [3]. Also, the use of artificial intelligence technology used alongside US may potentially assist with identifying anatomical structures and medical image interpretation [39].

Feedback revealed that the participants would like more sessions and more time per session which is mirrored in the literature [12, 18, 40]. Likewise, a study by Bullen et al. found that students sought additional and longer sessions with the US with smaller group sizes [18]. The student-to-demonstrator ratio should be kept small to motivate active participation and learning for all students. Learning in a small group setting enriches the learning and understanding of the subject [9]. Literature recommended that groups should consist of five (or fewer) students per US device, to allow all participants to observe the probe placement and on-screen images simultaneously [22]. Furthermore, for future sessions, careful thought should be provided to the scheduling and frequency of US sessions, to make sure that it strengthens the overall educational objective of the module.

### The Technical and Affective Experience of Using the US

Participants felt more confident in identifying anatomical structures as they become more familiar with the US [31]. This could be due to students’ improved knowledge of anatomy or with US, or both. The students were satisfied with the sessions, even though they found the anatomical structures difficult to identify due to inexperience with sonograms [12]. However, the process of learning to understand the orientation of the scan and the probe is significant as it will train students to comprehend complex 3D relations and spatial understanding between anatomical structures [8].

Motivating students to be active participants in their learning by using the US themselves made the session more relevant to the students. This form of contextualized learning is effective for adult learners to encourage a deeper understanding and promote the retention of facts [36]. The hands-on experience of teaching was perceived as enjoyable and educational as learning by doing is mostly regarded as an effective approach to learning [41]. By actively using the US, students are linking their textbook knowledge to the study of living anatomy to obtain views of dynamic regions, identify anatomical structures, and link deeper structures with surface anatomy [22]. Furthermore, converting a 2D view into a 3D spatial orientation (theme 2) involves the coordination of the visual and tactile senses [42]. These intricate coordinating skills may support memory retention (theme 4). These results resembled preceding studies where students found US to be an innovative approach and a successful way to teach anatomy [8, 18, 28].

## Conclusion

The research indicated that it is feasible and advantageous to include US sessions as an add-on to the teaching of anatomy during practical dissection sessions. The students embraced this novel tool and were actively engaged. This innovative, student-centered approach to anatomy teaching also adds to the rapidly evolving technological advances in health professions education. The use of innovative technologies, such as US, increases the interest of students, enables real-time viewing of dynamic anatomical relationships, enhances spatial ability, and allows students to gain skills and competencies in their learning process.

This study further emphasizes the importance of a multimodal teaching approach to optimize student learning, as students appear to learn more effectively when mixed educational methods and system-based approaches are integrated. Students appreciated the opportunity to correlate the cross-sectional US images together with the dissected or prosected specimens. The combination of the 2D US with the 3D cadaveric dissection or prosections enhanced their spatial awareness of anatomy. Feedback revealed that students would like more US sessions, more time, and more opportunities to be hands-on. The results of this study align with that of other institutions that have also run similar sessions in US.

US technology is added to human anatomy instruction in many medical sciences curricula. Anatomy teaching using US can act as a bridge, integrating anatomy learning and clinical practice by assisting students to apply anatomical knowledge. Nonetheless, these techniques have not routinely been transferred to anatomy modules for non-medical graduate-level courses. This study showed that clinical anatomy (non-medical) students connected the US sessions’ learning objective to their postgraduate interests and future careers.

## Limitations

The limitations of this study relate to the small sample size in one setting being used. The study was also restricted to teaching neck and thyroid anatomy in US, and it would be valuable to investigate if student perceptions of the use of US technology to learn the anatomy of different body regions would be just as positive. Another limitation was the amount of time available for the US session as the students spent only a short amount of time using the US. The study was also limited to students’ perceptions and did not measure if the students performed better academically after US sessions; this makes it difficult to deduce its value in terms of grades or assessment marks.

## Supplementary Information

Below is the link to the electronic supplementary material.

Supplementary file1 (DOCX 21 KB)

## Data Availability

The data that support the findings of this study are not openly available due to reasons of participant confidentiality, however are available from the corresponding author upon reasonable request.
