# Supplementary material for: Form and Function: Learning Anatomy Using Ultrasound
Source: Med Sci Educ. 2023 Jul 3;33(4):861–71. doi: 10.1007/s40670-023-01806-y (PMC10403465; doi:10.1007/s40670-023-01806-y)
Supplement: Supplementary file 1 — Supplementary file1 (DOCX 21 KB) [file 40670_2023_1806_MOESM1_ESM.docx]

# Supplementary Information 1

**VIRTUAL FOCUS GROUP SCHEDULE**

The virtual focus group interview schedule is outlined below:

1. Welcome and thanks for participating.
2. An overview of the topic.

Ultrasound use has expanded dramatically among medical specialities for diagnostic and interventional purposes, due to its affordability, portability, and practicality. This imaging modality, which permits real‐time visualization of anatomic structures and relationships in vivo, holds potential for undergraduate anatomy students.

Furthermore, living anatomy, as an educational component, is a neglected entity in anatomical sciences education. Students frequently find it demanding to visualize the functioning living anatomy of the human body therefore the implementation of ultrasound as an additional learning strategy may strengthen existing anatomical understanding and improve visual understanding of anatomy.

1. Statement of the ground rules of the virtual focus group, and assurance of confidentiality.
   1. Assurance of confidentiality
   2. Raise-hand's function, refer to students by initials
2. Start recorder.

The focus group questions/discussion (beginning with general experiences and progressing to specific problems).

1. How did you experience the ultrasound sessions during the dissection session?
2. What about the ultrasound sessions did you perceive as positive?
3. What about the ultrasound sessions did you perceive as negative? How can we improve the sessions?
4. How did you find the pre-ultrasound material that was posted on SUNLearn?
5. Cross-sections are two-dimensional views of gross anatomical structures in transverse planes. How do you think ultrasound benefited (or not) your learning of cross-sectional anatomy?
6. How do you feel about using ultrasound as an add-on during dissections to study living anatomy?
7. How did you find the identification of the anatomical structures while using the ultrasound?
8. Do you think using ultrasound will create a link between undergraduate anatomy and clinical relevance?
9. How did you experience the hands-on involvement of the ultrasound session?
10. Do you have any closing statements on ultrasound and its use in your teaching and learning anatomy?
11. Stop recorder.
